# Primary mediastinal hemangiopericytoma

**DOI:** 10.1186/1477-7819-4-23

**Published:** 2006-04-27

**Authors:** A Chnaris, N Barbetakis, A Efstathiou, I Fessatidis

**Affiliations:** 1 Cardiothoracic Surgery Department, G. Papanikolaou General Hospital, Thessaloniki, Greece; 2 Cardiothoracic Surgery Department, Theagenio Cancer Hospital, Thessaloniki, Greece; 3 Cardiothoracic Surgery Department, Geniki Kliniki, Thessaloniki, Greece

## Abstract

**Background:**

Hemangiopericytoma is a rare mesenchymal neoplasm, accounting for about 1% of vascular tumors The tumor occurs most commonly in the skin, subcutaneous soft tissues, muscles of the extremities, retroperitoneum but rarely in the lung, trachea or mediastinum.

**Case presentation:**

A rare case of primary mediastinal hemangiopericytoma is presented. A 72-year-old woman was treated by complete surgical resection of the tumor. Details of the clinical and radiographic feature are presented. The patient's postoperative course was uneventful with no evidence of recurrence 9 months after the operation.

**Conclusion:**

Hemangiopericytoma is an uncommon, potentially malignant tumor originating from pericytes in the small vessels and surgical radical excision is the treatment of choice, although the criteria for determining the area of resection have not been established. International literature has demonstrated that recurrent disease usually occurs within 2 years and therefore a long-term careful follow-up is required.

## Background

Hemangiopericytoma is a rare mesenchymal neoplasm, accounting for about 1% of vascular tumors [1]. Hemangiopericytoma is known to be derived from the vascular pericyte and was first reported by Stout and Murray in 1942 [2]. The tumor occurs most commonly in the skin, subcutaneous soft tissues, muscles of the extremities, retroperitoneum but rarely in the lung, trachea or mediastinum [3]. Herein, a surgical case of primary mediastinal hemangiopericytoma is presented.

## Case presentation

**Figure 1:**
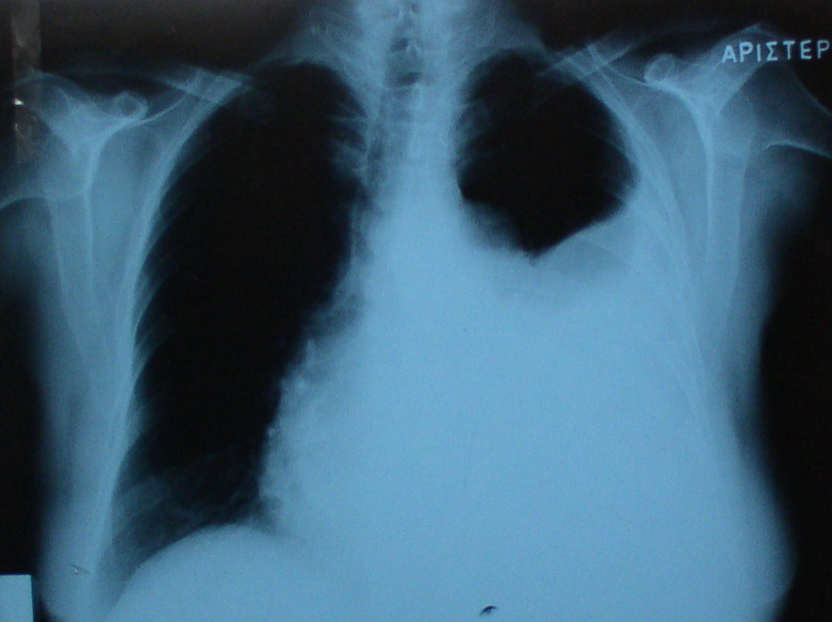
Chest X-ray showing an abnormal shadow associated with ipsilateral pleural effusion.

**Figure 2:**
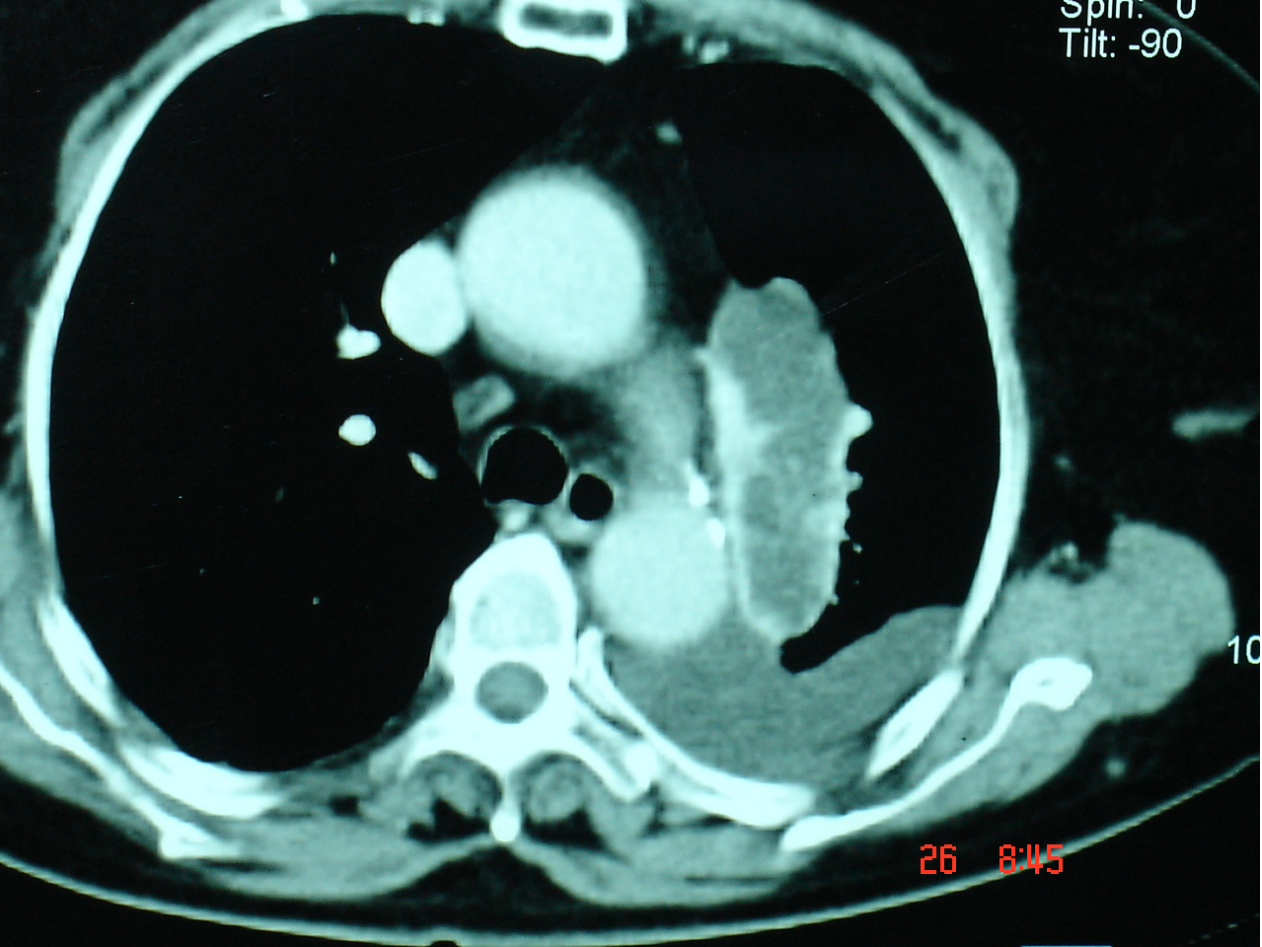
Preoperative computed tomographic scan demonstrated a tumor mass located in the mediastinum next to descending aorta and associated with pleural effusion.

A 72-year-old woman was referred to our institution complaining of dyspnea, cough and chest tightness for the last two months. Physical examination indicated tachypnea, orthopnea and use of accessory respiratory muscles. Laboratory studies were essentially within normal limits. A chest X-ray revealed a homogenous opacity occupying lower two-thirds of the left hemithorax and causing contralateral shifting of the mediastinum and heart (Figure 1). Thoracentesis was performed in order to palliate respiratory problems. One thousand eight hundred (1800) ml of serosanguinous fluid were aspirated. Cytologic examination revealed an exudate with no signs of malignancy. Culture of the aspirated pleural fluid was also negative. A computed tomographic scan (CT) of the chest revealed a large solid tumorous mass measuring 7.3 × 2.3 cm located in the posterosuperior mediastinum on the left side associated with pleural effusion (Figure 2). In order to perform preoperative staging of the tumor, the patient underwent CT scans of brain, upper abdomen, a bone scan and broncoscopy. All were normal. The tumor markers alpha-fetoprotein (AFP), carcinoembryonic antigen (CEA), CA 19-9, neuron-specific enolase (NSE) and squamous cell carcinoma antigen were within normal limits. The mass seemed to be resectable and surgical approach was suggested to the patient.

**Figure 3:**
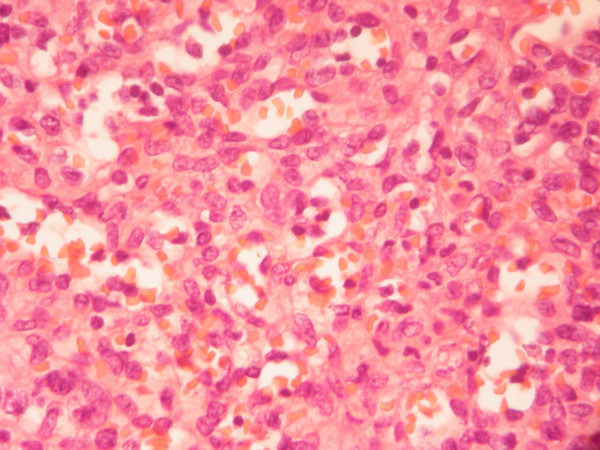
Round and spindle cells surrounded by thin-walled, endothelium-lined vascular channels, giving a "staghorn" appearance to the vessels as typically seen in hemangiopericytoma (Hematoxylin and eosin stain ×400).

**Figure 4:**
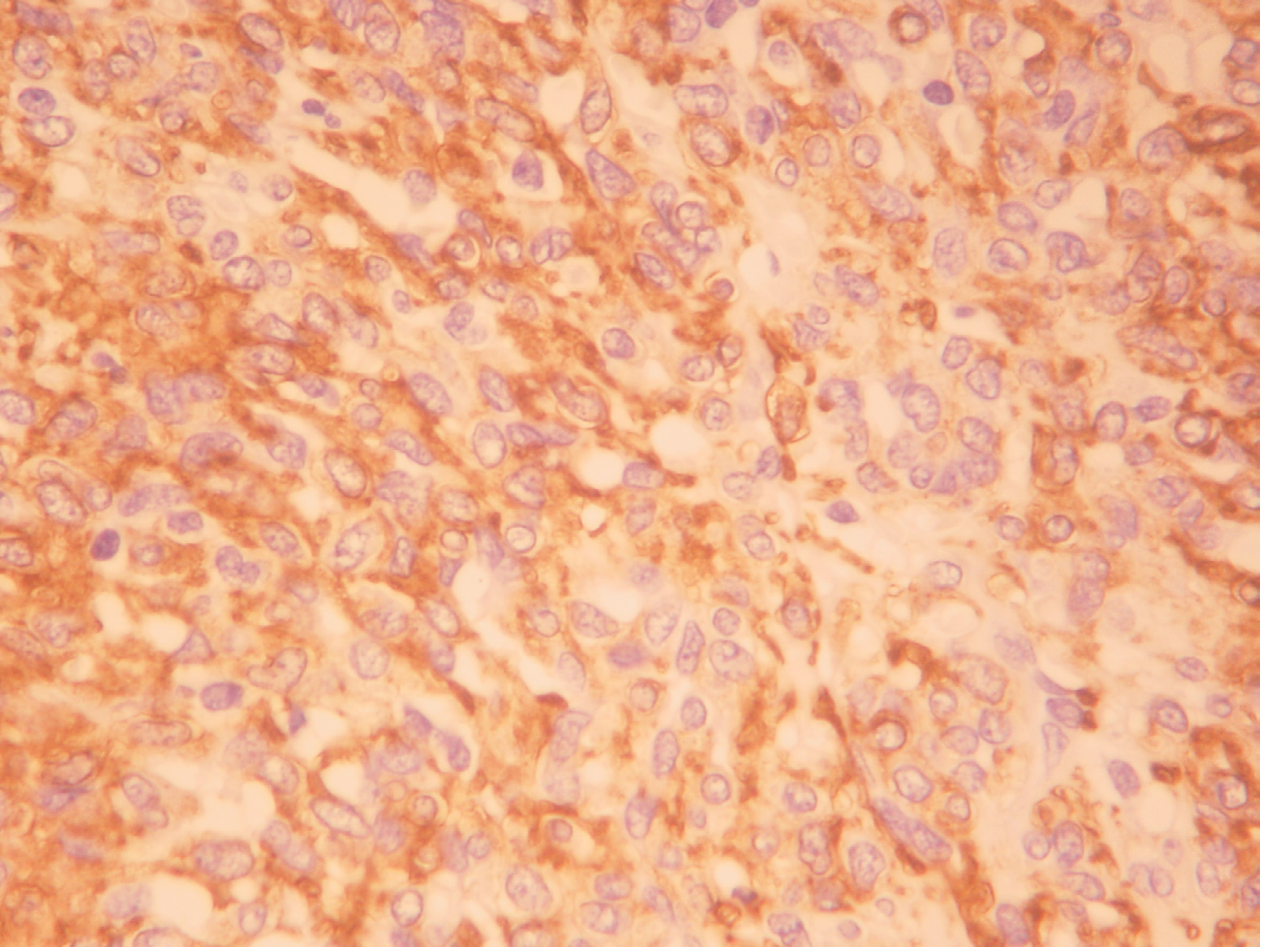
Tumor cells clearly positive for a-SMA (anti-smooth-muscle-actin) protein (×400).

The patient underwent left posterolateral thoracotomy through 5^th ^intercostal space. During the operation, the mass appeared to have mediastinal origin and was highly vascularized. There was no involvement of vital mediastinal structures and the surrounding lung parenchyma was compressed by the tumor. Six hundred ml of sanguinous pleural fluid were aspirated. Despite the persistent bleeding during dissection the tumor was resected. The mass measured 7 × 3 × 2.5 cm and weighed 210 g. Grossly the resected specimen was a smooth, friable encapsulated mass with focal hemorrhages. The cut surface was smooth, elastic and pale brown. Microscopic examination showed round and spindle cells surrounded by thin-walled, endothelium-lined vascular channels, giving a "staghorn" appearance to the vessels as typically seen in hemangiopericytoma (Figure 3). The low-mitotic activity and the absence of clear nuclear pleomorphism were suggestive of a low-grade malignant tumor. The tumor cells were immunoreactive only for anti-smooth-muscle-actin protein (Figure 4).

The postoperative course was uneventful and the patient was discharged home on postoperative day 9. Postoperative chemoradiotherapy was recommended but the patient denied any further treatment. Nine months after the operation the patient is alive and well without evidence of recurrence of the disease.

## Discussion

Hemangiopericytoma is an uncommon, potentially malignant tumor originating from pericytes in the small vessels. Intrathoracic hemangiopericytoma usually arises from pericytes that surround the basement membrane of capillaries and small venules within the lung parenchyma [3]. Our case was an intrathoracic mediastinal hemangiopericytoma, which is extremely rare. Only a few isolated case reports are available in the literature [4-7], whereas the intrapulmonary variety of the same tumor is relatively more common.

Hemangiopericytoma has no uniform clinical or radiographic features, usually affects older individuals, and mostly presents as an asymptomatic, non-calcified solitary mass on chest X-ray. These tumors are composed of closely-packed spindle cells and prominent vascular channels. The histological differential diagnosis includes many mesenchymal tumors, such as the solitary fibrous tumor and the synovial sarcoma [3]. No single clinical or histological feature including histological type or DNA ploidy allows prediction of biologic aggressiveness [8]. Malignant hemangiopericytoma is recognized by its increased mitotic rate, tumor size and foci of hemorrhage and necrosis [3].

Immunohistochemically, hemangiopericytomas are known to show a positive response to antibodies against vimentin and type IV collagen and a negative response to VIII-related antigen, S-100 protein, neuron specific enolase, carcinoembryonic antigen, desmins, laminin and cytokeratins [9].

A special consideration concerns the preoperative diagnosis. When a mass appears to be radiologically resectable, many authors perform a thoracotomy without histological diagnosis. Previous reports propose an attempt to obtain a preoperative diagnosis even in tumors that are clearly resectable if high vascularization is suspected on imaging techniques [10,11].

Surgical radical excision is the treatment of choice for hemangiopericytomas, although the criteria for determining the area of resection have not been established. Hansen and colleagues stated that it was necessary to consider all hemangiopericytomas as malignant and perform extended surgery [12]. During the resection, it is important to look for invasion of the surrounding lung tissue and to avoid intrathoracic spread of tumor cells by manual examination. With respect to adjuvant therapy, chemotherapy or radiotherapy have been recommended but is considered to be almost ineffective [3]. On the other hand Rusch *et al*., reported that combination therapy or single therapy with adriamycin was effective against metastases [13]. Jalal and Jeyasingham reported that preoperative radiotherapy of large hemangiopericytomas on the chest wall significantly reduced the vascularity of the tumor and made complete resection much easier [14]. Some authors have proposed an innovative approach of treatment, which includes complete surgical resection along with intraoperative and postoperative radiotherapy, whereas others have recommended that radiotherapy may be used palliatively for local tumor recurrence or superior vena cava obstruction [13,15,16]. Morandi *et al*., recommended preoperative percutaneous embolization of hypervascular mediastinal tumors, in order to allow a safe complete removal of the lesion later [17].

The 5-year survival of patients with hemangiopericytoma originating in any organ has been reported to be 85%, whereas the survival of patients with a tumor of pulmonary origin is 30–35%. Approximately 50% of hemangiopericytomas have been reported to recur within 5 years [3,12]. It has been demonstrated that recurrent disease usually occurs within 2 years after initial treatment and recurrences are commonly found in the thorax, either in the pulmonary parenchyma or in the pleura. Distant metastases to liver, brain and bone have also been reported [12].

## Conclusion

Hemangiopericytoma is rare vascular slow-growing tumor with high local recurrence and the long-term prognosis is poor because of its propensity to recur. Surgical radical excision is the treatment of choice despite the fact that the risk of intraoperative uncontrollable bleeding is high. Local or distant recurrence is commonly seen and a long-term careful follow-up is required.

## Competing interests

The author(s) declare that they have no competing interests.

## Authors' contributions

AC, NB, AE took part in the care of the patient and contributed equally in carrying out the medical literature search and preparation of the manuscript. IF participated in the care of the patient and had the supervision of this report. All authors approved the final manuscript.

## References

[B1] Hart LL, Weinberg JB (1987). Metastatic hemangiopericytoma with prolonged survival. Cancer.

[B2] Stout AP, Murray MR (1942). Hemangiopericytoma: a vascular tumor featuring Zimmerman's pericytes. Ann Surg.

[B3] Espat NJ, Lewis JJ, Leung D (2002). Conventional hemangiopericytoma: modern analysis of outcome. Cancer.

[B4] Simonton SC, Swanson PE, Watterson J, Priest JR (1995). Primary mediastinal hemangiopericytoma with fatal outcome in a child. Arch Pathol Lab Med.

[B5] Hayashi A, Takamori S, Tayama K, Mitsuoka M, Tamura K, Shirouzu K, Fujimoto K, Watanabe J (1998). Primary hemangiopericytoma of the superior mediastinum: a case report. Ann Thorac Cardiovasc Surg.

[B6] Mori M, Nakanishi N, Furuya K (1994). Hemangiopericytoma of the mediastinum causing spontaneous hemothorax. Ann Thorac Surg.

[B7] Gomez Finana MS, Paya Perez L, Parede Osaelo JR, Aranda Lopez I, Massuti Sureda B, Talavera Sanchez J (1994). Hemangiopericytoma of the soft palate and mediastinum: a case report. Acta Otorrinolaringol Esp.

[B8] Fukunaga M, Shimoda T, Nikaido T, Ushigom S, Ishikawa E (1993). Soft tissue vascular tumor. A flow cytometric DNA analysis. Cancer.

[B9] Yoshida M, Morita M, Kakimoto S, Kawakami M, Sasaki S (2003). Primary hemangiopericytoma of the trachea. Ann Thorac Surg.

[B10] Baldo X, Sureda C, Gimferrer JM, Belda J (1997). Primary mediastinal leiomyoma. Eur J Cardiothorac Surg.

[B11] Fiumara E, D'Angelo V, Florio FP, Nardella M, Biscelia M (1996). Preoperative embolization in surgical treatment of spinal thoracic dumpbell schwannoma. J Neurosurg Sci.

[B12] Hansen CP, Francis D, Bertelsen S (1990). Primaryhemangiopericytoma of the lung. Scand J Thorac Cardiovasc Surg.

[B13] Rusch VW, Shuman WP, Schmidt R, Laramore GE (1989). Massive pulmonary hemangiopericytoma. An innovative approach to evaluation and treatment. Cancer.

[B14] Jalal A, Jeyasingham K (1999). Massive intrathoracic extrapleural hemangiopericytoma: deployment of radiotherapy to reduce vascularity. Eur J Cardiothorac Surg.

[B15] Mira JG, Chu FCH, Fortner JG (1975). The role of radiotherapy in the management of malignant hemangiopericytoma – report of 11 cases and review of the literature. Cancer.

[B16] Jha N, McNeese M, Barkley HT, Kong J (1987). Doesradiotherapy have a role in hemangiopericytoma management?. Int J Radiat Oncol Biol Phys.

[B17] Morandi U, Stefani A, De Santis M, Paci M, Lodi R (2000). Preoperative embolization in surgical treatment of mediastinal hemangiopericytoma. Ann Thorac Surg.

